# Enhanced Directed Random Walk for the Identification of Breast Cancer Prognostic Markers from Multiclass Expression Data

**DOI:** 10.3390/e23091232

**Published:** 2021-09-20

**Authors:** Hui Wen Nies, Mohd Saberi Mohamad, Zalmiyah Zakaria, Weng Howe Chan, Muhammad Akmal Remli, Yong Hui Nies

**Affiliations:** 1 School of Computing, Faculty of Engineering, Universiti Teknologi Malaysia, Skudai 81310, Malaysia; zalmiyah@utm.my (Z.Z.); cwenghowe@utm.my (W.H.C.); 2 Health Data Science Lab, Department of Genetics and Genomics, College of Medical and Health Sciences, United Arab Emirates University, Al Ain 17666, United Arab Emirates; saberi@uaeu.ac.ae; 3 Institute for Artificial Intelligence and Big Data, Universiti Malaysia Kelantan, Kota Bharu 16100, Malaysia; akmal@umk.edu.my; 4 Department of Anatomy, Faculty of Medicine, Universiti Kebangsaan Malaysia Medical Centre, Cheras, Kuala Lumpur 56000, Malaysia; p100341@siswa.ukm.edu.my

**Keywords:** prognostic markers, breast cancer, multiclass, microarray analysis, ANOVA, pathway selection, directed random walk

## Abstract

Artificial intelligence in healthcare can potentially identify the probability of contracting a particular disease more accurately. There are five common molecular subtypes of breast cancer: luminal A, luminal B, basal, ERBB2, and normal-like. Previous investigations showed that pathway-based microarray analysis could help in the identification of prognostic markers from gene expressions. For example, directed random walk (DRW) can infer a greater reproducibility power of the pathway activity between two classes of samples with a higher classification accuracy. However, most of the existing methods (including DRW) ignored the characteristics of different cancer subtypes and considered all of the pathways to contribute equally to the analysis. Therefore, an enhanced DRW (eDRW+) is proposed to identify breast cancer prognostic markers from multiclass expression data. An improved weight strategy using one-way ANOVA (F-test) and pathway selection based on the greatest reproducibility power is proposed in eDRW+. The experimental results show that the eDRW+ exceeds other methods in terms of AUC. Besides this, the eDRW+ identifies 294 gene markers and 45 pathway markers from the breast cancer datasets with better AUC. Therefore, the prognostic markers (pathway markers and gene markers) can identify drug targets and look for cancer subtypes with clinically distinct outcomes.

## 1. Introduction

Cancer is associated with abnormal alterations that lead to the dysregulation of the cellular system [1]. Breast cancer is the most common cancer found in women worldwide [2]. Luminal A, luminal B, basal, ERBB2, and normal-like are the five molecular subtypes of breast cancer from gene expression profiling. Besides this, the accurate classification of diseases and treatment responses is helpful in clinical and cancer biology research [1,3,4]. The classification aims to identify patients with similar clinical features (characteristics) in order to identify and implement suitable treatments [5]. Pathway-based microarray analysis reduces its complexity of analysis from thousands of genes to a few hundred pathways [6]. However, most of the existing methods, such as principal component analysis [PCA] in combination with agglomerative hierarchical clustering [AHC] [7], mean-centering, and magnitude normalization [8], only use gene expression data for microarray analysis. Other than that, directed random walk (DRW) is one of the pathway-based microarray analyses that uses gene expression data, pathways, and directed graphs. It exploits pathway and topology information to infer a greater reproducibility power of pathway activity between normal and disease samples, together with a weighting strategy (using t-test statistics with equal variances) [4,9,10]. 

In literature, some existing pathway-based microarray analyses are restricted to binary class classification [11], including DRW [4]. For example, negatively correlated feature sets with ideal markers (NCFS-i) and negatively correlated feature sets with condition-responsive genes (NCFS-CORG) methods use a t-test to infer the pathway activities between relapse and non-relapse samples [12,13,14]. However, the t-test is typically used in only two classes of samples [15,16]. Some studies modify the t-test to deal with multiclass problems [10,17,18]. In most of the literature, an analysis of variance (ANOVA) F-test statistic is frequently used to solve multiclass issues [19,20,21,22]. Besides this, multiclass classification methods can be divided into two types [23]. The first, involves extending the binary classification to deal with multiclass problems directly [23,24]. The other type involves decomposing multiclass issues into binary issues [23]. One-versus-one and one-versus-the-rest are common strategies for dealing with multiclass problems [25]. However, some of the existing binary methods are not extensible to multiclass approaches [24]. Recent medical studies reported the necessity of the diagnosis of more than two classes of disease [11,17,22,26]. In this case, pathway data can allow us to better understand molecular mechanisms based on cancer subtypes [27].

Several studies in pathway-based microarray analysis did not select pathways [4,12,13,14,28]. Because the pathways were commonly curated from the literature, non-informative genes could also be included [29,30]. If a gene is selected, all of the pathways consisting of the gene will also be chosen [28]. The presence of non-informative data can affect the accuracy of the methods [31]. Thus, pathway selection can reduce the dimensions and select informative pathways in all examples [25]. In most cases, pathway selections are performed to remove redundancy in the pathway-based feature selection [25,28]. In general, these selection methods are based on statistical tests like the t-test and Fisher-test [25].

**Figure 1:**
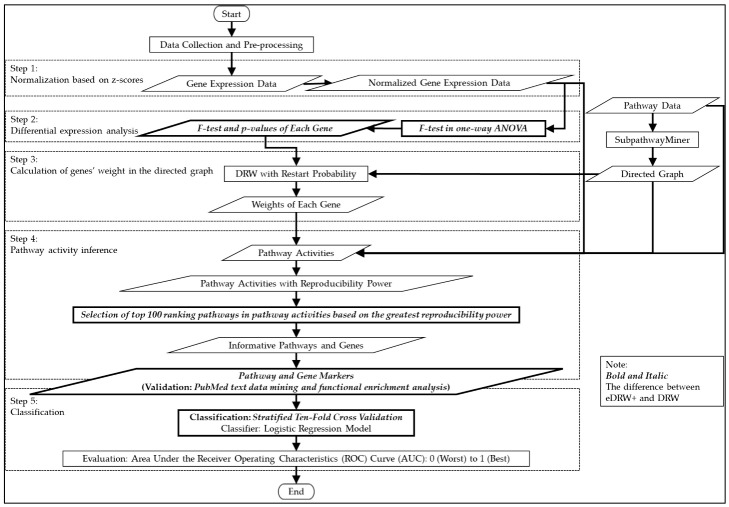
Flowchart of the eDRW+.

Here, an enhanced DRW (eDRW+) is proposed to identify breast cancer prognostic markers from multiclass pathway expression data. An improved weight strategy using one-way ANOVA (F-test) and pathway selection based on the greatest reproducibility power is proposed in eDRW+. The ANOVA used in eDRW+ is intended to identify differentially expressed genes for multiple cancer subtypes. Hence, the weight of the genes is also essential to identify informative genes in the directed graph. Subsequently, the pathway selection used in eDRW+ is intended to select the top 100 ranking pathways in the pathway activities based on the highest reproducibility power, increasing the method’s classification performance. The proposed method is implemented in the R platform with version 3.3.3 in 64-bit using Windows 10. Figure 1 shows the flowchart of eDRW+. The overall eDRW+ includes normalization based on z-scores, differential expression analysis, the calculation of the genes’ weight in the directed graph, the inference of the pathway activities, and classification.

## 2. Materials and Methods

### 2.1. Data Collection and Pre-Processing

**Figure 2:**
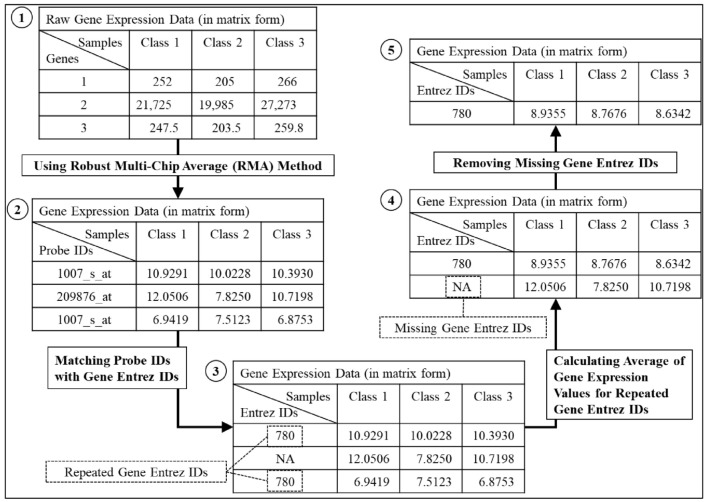
The flow of the pre-processing step for the gene expression data.

The input data used are gene expression data, pathway data, and a directed graph. The gene expression data were downloaded from NCBI’s Gene Expression Omnibus (GEO) repository (breast cancers: GSE1456 and GSE1561). This data was identified using microarray analysis [32,33]. GSE1456 is the dataset collected from all of the breast cancer patients who received surgery at Karolinska Hospital between 1994 and 1996 [33]. For GSE1561, there is a phase III clinical trial dataset, but the clinical response data is not yet available [32]. GSE1456 and GSE1561 are the gene expression data commonly used for multiclass classifications that cover different types of disease [11]. The dataset samples comprise five biological groups: apocrine, basal, ERBB2, luminal, and normal. Because the raw gene expression data consist of missing and repeated gene Entrez ID, dataset pre-processing was performed. Missing and repeated data can lead to poor survival analysis and the incorrect interpretation of predictors like the diagnosis stage [34]. Figure 2 shows the flow of the pre-processing process on the gene expression data. Based on [35,36], the missing gene Entrez IDs were removed, and the gene expression values of the repeated gene Entrez IDs were averaged across all of the samples. Table 1 presents the details of the gene expression data used in this research. 

A total of 300 pathways, including 150 metabolic pathways and 150 non-metabolic pathways, were collected from the Kyoto Encyclopedia of Genes and Genomes (KEGG) database [4]. The pathways and directed graph were downloaded from the R package *DRWPClass*. The directed graph covered 4113 genes and 40,875 directed edges (interaction between genes) [4]. The types of interaction between the genes, weight, and position of the genes were the general topological features of the directed graph.

Regarding the directed graph, the types of interaction between the genes reflected the ways in which the genes interacted with and regulated each other. Hence, the direction of the edges can be extracted from the KEGG database. For example, gene Entrez ID 11260 points to gene Entrez ID 5901 in the directed graph, which can be derived from XPOT (gene Entrez ID: 11260) inhibiting RAN (gene Entrez ID: 5901) in the RNA transport (KEGG pathway ID: hsa03013).

### 2.2. Step 1: Normalization Based on Z-Scores

Normalization based on z-scores is used to standardize the gene expression values over all of the samples (different biological conditions) to a scale of mean zero and variance one [4,37,38]. The formula of the normalization based on z-scores was as it is given below:

(1) $$z\left(g_{i}\right)=\;\left(gene\left[i\right]-\bar{X}\left[i\right]\right)\;/\;S\left[i\right]$$

where *z(g_i_)* is the normalized gene expression value for gene *i* over all of the samples, *gene*[*i*] is the gene expression value for gene *i* over all of the samples, $\bar{X}$[*i*] is the mean of the gene expression values for gene *i*, *S*[*i*] is the standard deviation of the gene expression values for gene *i*, and *i* is the number of genes in the gene expression data.

### 2.3. Step 2: Differential Expression Analysis

**Figure 3:**
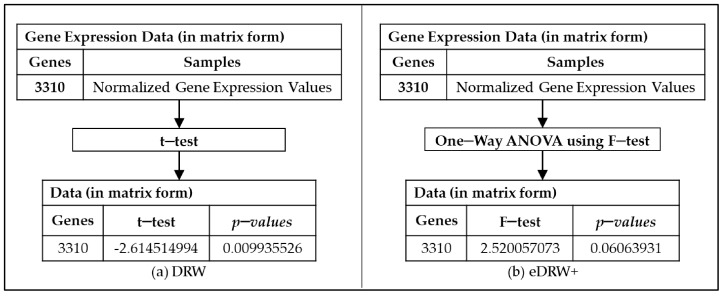
Comparison of the statistical tests employed in DRW (**a**) and eDRW+ (**b**).

One-way ANOVA using an F-test is used in the proposed method eDRW+ instead of the t-test, which improves the weight of the genes to show their importance in the directed graph. A comparison of the statistical tests employed in DRW and eDRW+ is illustrated in Figure 3a,b. A high F statistic indicates a significant difference between the group’s averages. The group meant multiple classes of samples reflected as an independent variable to show that the genes are differentially expressed, and the gene expression data is a dependent variable. One-way ANOVA (F-test) is commonly used to assess normalized gene expression level variances and the mean difference between classes [15,22,39,40]. The combination of ANOVA and an F-test is significant in comparisons and experiment error rates [41]. Based on Lix et al. [42], a considerable amount of literature demonstrated the robustness of using ANOVA to support the F-test in most data analytic situations. Hence, the F-test was used to solve multiclass problems and calculate the weight of the genes and the reproducibility power of pathway activities. The formula for the F-test used in the differential expression analysis is shown below:

(2) $$F_{test}\left(g_{i}\right)=\frac{\left(\sum_{i=1}^{i}\frac{{\left(\sum_{i=1}^{i}z\left(g_{i}\right)\right)}^{2}}{n}\right)-(\frac{{\left(\sum_{i=1}^{i}z\left(g_{i}\right)\right)}^{2}}{n})/\left(k-1\right)}{\left(\sum_{i=1}^{i}z{\left(g_{i}\right)}^{2}-\left(\frac{{\left(\sum_{i=1}^{i}z\left(g_{i}\right)\right)}^{2}}{n}\right)\right)-\left(\left(\sum_{i=1}^{i}\frac{{\left(\sum_{i=1}^{i}z\left(g_{i}\right)\right)}^{2}}{n}\right)-\left(\frac{{\left(\sum_{i=1}^{i}z\left(g_{i}\right)\right)}^{2}}{n}\right)\right)/\left(N-k\right)}$$

where *i* is the number of genes, *z(g_i_)* is the normalized gene expression value for each gene (obtained from Equation (1)), *n* is the number of samples for each class, *k* is the total number of classes, and *N* is the total number of samples for all of the classes.

### 2.4. Step 3: Calculation of the Genes’ Weight in the Directed Graph

**Figure 4:**
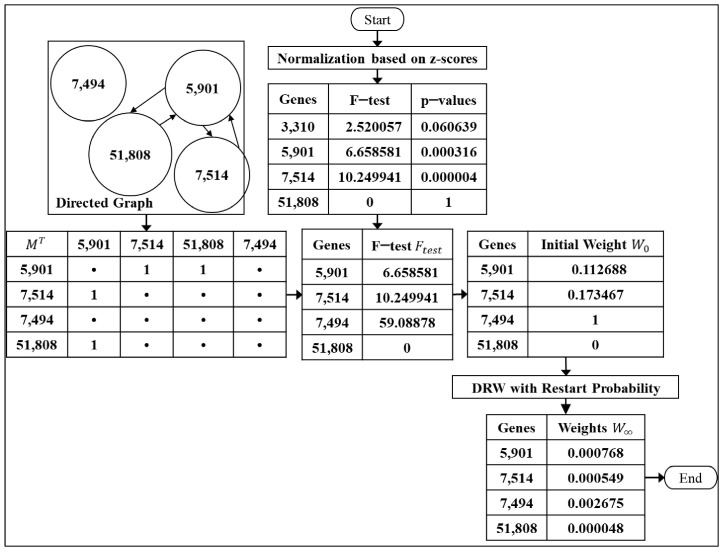
The calculation of the genes’ weight for eDRW+.

We the proceed to calculate the weight of the genes after the differential expression analysis; the weight of the genes is further used for the pathway activity inference. Figure 4 shows the calculation of the genes’ weight for eDRW+. The F-test and *p*-values were calculated in Equation (2) in order to calculate the initial weight. The initial weight of the genes was used as a vector that held the probability at the specific node. The directed graph was converted to an adjacency matrix and combined with the virtual ground nodes. Then, we proceeded to calculate the edge weight of the genes in the directed graph for eDRW+. The restart probability (*r*) was the only parameter set to 0.7, the same as DRW [4]. Note that the *r* values did not significantly change in the area under the receiver operating characteristics curve (AUC). The formula for the calculation of the initial weight of the genes is shown below:

(3) $$W_{0}=\frac{absolute\;F_{test}-maximum\;F_{test}}{maximum\;F_{test}-minimum\;F_{test}}$$

where $W_{0}$ denotes the initial weight of the genes, $absolute\;F_{test}\;$is the absolute value of the F-test statistic, $maximum\;F_{test}$ is the maximum value of the F-test statistic, $minimum\;F_{test}$ is the minimum value of the F-test statistic, and $F_{test}$ is obtained from the formula of the F-test in Equation (2).

In order to calculate the edge weights in the directed graph, the theory of random walk used by DRW and eDRW+ remains the same. DRW with the restart probability is defined as:

(4) $$DRW\;with\;restart\;probability,\;W_{t+1}=\left(1-r\right)M^{T}W_{t}+rW_{0}$$

where r denotes the restart probability (set to 0.7), M denotes the row-normalized adjacency matrix of a directed graph, e.g., the adjacency matrix represented the interaction between two genes in the directed graph, and the weight is 1 when the interaction exists, otherwise is 0, $W_{t}$ is a vector that holds the probability at the specific node at a time step t, e.g., the random walk starts a node at t = 0, then it randomly points to the second node at t = 1, until no nodes are indicated at the last time step, the nodes have been visited at the previous time step that cannot be revisited at the next time step, and $W_{0}$ is the initial weight of the genes (obtained from Equation (3)).

### 2.5. Step 4: Inferring Pathway Activities

**Figure 5:**
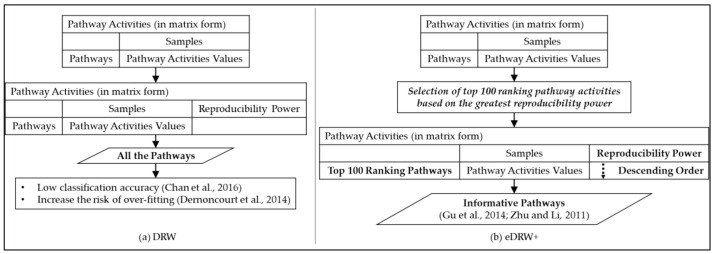
Difference between DRW (**a**) and eDRW+ (**b**) in the selection of the pathways.

**Figure 6:**
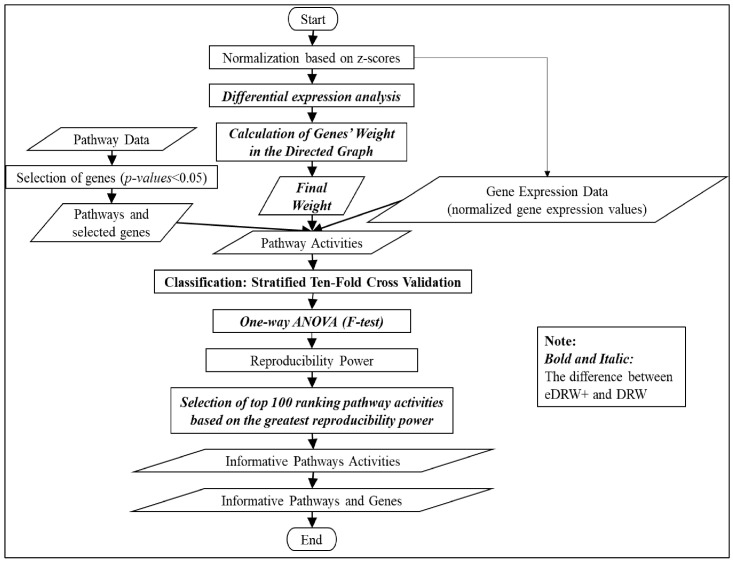
Overview of the pathway activity inference and pathway selection.

For each pathway in the pathway data, those genes with *p*-values less than 0.05 are selected to construct the pathway activities [4]. Figure 5 presents the difference between DRW (a) and eDRW+ (b), without and with the selection of the pathways. Because the pathways were typically collected from a curated community database, the presence of non-informative data can lead to low classification accuracy and increase the risk of over-fitting [30,43,44,45,46]. Therefore, the selection of the top 100 ranking pathways in pathway activities has been proposed, based on the greatest reproducibility power. Figure 6 shows an overview of the pathway activity inference. The formula used to measure the pathway activity for both the training and test sets is shown below:

(5) $$a\left(P_{j}\right)=\frac{\sum_{i=1}^{n_{j}}W_{\infty }\left(g_{i}\right)\cdot sgn\left(F_{score}\left(g_{i}\right)\right)\cdot z\left(g_{i}\right)}{\sqrt{\sum_{i=1}^{n_{j}}{\left(W_{\infty }\left(g_{i}\right)\right)}^{2}}}$$

where $a\left(P_{j}\right)$ denotes the pathway activity (or expression value), $W_{\infty }$ is the weight of the genes (obtained from Equation (4)), $F_{score}\left(g_{i}\right)$ is the F-test statistic of gene $g_{i}$ from the one-way ANOVA on expression values between multiple classes of samples, e.g., the F-test of each gene is obtained from Equation (2), and then it is also used to show the statistical difference of the gene between the classes; $P_{j}\;$denotes the pathways in row j, e.g., 300 pathways are used; row i of the gene profiles $z\left(g_{i}\right)$ is the expression value of gene $g_{i}$ across the entire dataset (obtained from Equation (1)); and sgn() is the sign function, which returns −1 for negative numbers and +1 for positive numbers.

The reproducibility power of the pathway activity reflected the training and test sets to show its discriminative power and robustness [4,43]. The greater reproducibility power of a pathway activity defines the pathway activity with more discriminative power and stronger robustness. The reproducibility power is shown as follows:

(6) $$C_{score}\left(N\right)=\frac{1}{N}\sum_{i=1}^{N}F_{score}\left(P_{T}^{i}\right)\cdot F_{score}\left(P_{V}^{i}\right)$$

where $F_{score}\left(P\right)$ is the F-test statistics of P from the one-way ANOVA on the pathway activities between multiple classes of samples, e.g., using Equation (2) to calculate the F-test for each pathway; $P_{T}^{i}$ is the i-th pathway activity over the top 100 pathways of the pathway activities in descending order (ranked by absolute F-test statistics) in the training dataset, e.g., 300 pathways used; the genes with *p*-values < 0.05 have been selected only for each pathway, but not all of the pathways consisted of such genes, such that a total of 240 pathways are left in this step, so a selection of the top 100 pathways has only been proposed based on the greatest values of the F-test; $P_{V}^{i}$ is its corresponding pathway activities in the test dataset, e.g., the informative pathways in the dataset consisting of all of the sample classes; and N is the number of selected pathways, e.g., the informative pathways.

### 2.6. Step 5: Classification

The classification performances are evaluated to identify the cancer outcomes in a stratified ten-fold cross-validation. In doing so, this cross-validation was carried out within and between the datasets [43]. For the stratified ten-fold cross-validation within datasets, the training and test sets consisted of the same data. The samples in a dataset were randomly divided into ten subsets of equal size. Each fold consisted of roughly the same proportion of the class labels. Eight subsets were used as the training samples to develop the model, with the ninth subset as the validation sample, and the tenth subset as the testing sample. For the stratified ten-fold cross-validation between the datasets, the first entire dataset was used as the training set, and the second independent dataset was used as the test set [4,43]. The first dataset was divided into ten subsets. Nine subsets were used to train the classifier, while the remaining one was used to optimize the constructed classifier and select the best feature set. The cross-validation process is repeated ten times (the folds), with each of the ten subsamples being used exactly once, as the validation data. The procedure was then repeated ten times, and the results were averaged.

A logistic regression model, a support vector machine (SVM), and Naïve Bayes were used as the classifiers to train and test the pathway activity. A performance measure can be computed based on the area under the receiver operating characteristic curve (AUC). An AUC closer to 1.0 indicates a more accurate classification, while an AUC closer to 0.5 indicates a worse classification [4,22,47].

The informative pathways and genes identified by eDRW+ were then analyzed using PubMed text data mining [http://www.ncbi.nlm.nih.gov/pubmed?LinkName=gene_pubmed&from_uid=2066] (accessed on 23 July 2021) and the Functional Annotation tool from the Database for Annotation, Visualization, and Integrated Discovery (DAVID) [https://david.ncifcrf.gov/summary.jsp] (accessed on 3 August 2021). PubMed text data mining is used to show the relationship between pathways, genes, and cancers [47,48,49]. “Pathway names,” “gene names,” “breast cancer”, “breast adenocarcinoma”, and “breast carcinoma” were selected as the concepts for extraction. “Prognostic” and “cancer marker” were the keyword terms employed to show the pathways and genes exhibiting biological characteristics related to the cancers. PubMed identifiers (PMIDs) were obtained as evidence to ascertain the relationship between pathways, genes, and diseases [48,49]. For the Functional Annotation Tool, it is helpful to annotate the identified pathways and genes based on the Gene Ontology and KEGG databases [48,50,51,52,53]. Fisher’s exact test is used in the tool to measure the gene-enrichment in annotation terms. This enrichment can help identify the proportions of different gene groups falling into one or two mutually exclusive categories. The gene list has been annotated to the KEGG and OMIM databases.

Table 2 summarizes the comparison between DRW and eDRW+. Several classification methods from the literature have been implemented to compare eDRW+’s classification results. These methods use either gene expression data only, or both gene expression data and pathways in the microarray analysis. Principal component analysis (PCA) combined with agglomerative hierarchical clustering (AHC) [7] and mean-centering and magnitude-normalization [8] are the gene-based classification methods used in binary class classification. Negatively correlated feature sets with ideal markers (NCFS-i) [12,14,17] and negatively correlated feature sets with condition-responsive genes (NCFS-CORG) [13] methods are the pathway-based classification methods for the inference of the pathway activities between two classes. NCFS-i and NCFS-CORG applied a logistic regression model and Naïve Bayes to build the classifiers.

## 3. Results

The results were collected from ten runs of an experiment in a stratified ten-fold cross-validation within and between datasets based on breast cancer (GSE1456 and GSE1561). Previous researchers have successfully analyzed the GSE1456 and GSE1561 datasets to link the identified pathways and genes with breast cancer, with high prediction accuracies [11]. Besides this, the multicollinearity analysis of the studied dataset has been applied to check the ways in which the correlation exists when different samples are used, or when additional variables are added. Several classification methods from the literature have been implemented to compare eDRW+’s classification results.

### 3.1. Multicollinearity Analysis

The *mctest()* function in the R *mctest* package has been applied to detect the multicollinearity diagnostic measures on the studied datasets. Multicollinearity analysis can also avoid multicollinearity problems [54]. This problem can result from the repetition of the same variable. For example, the variables are highly correlated with each other. The presence of multicollinearity can influence the power of statistical significance (e.g., p-values). The variables of multicollinearity diagnostics are the variance inflation factor (VIF), tolerance limit (TOL), Leamer’s method, Red indicator, and $R^{2}$.

Because the raw GSE1456 dataset was collected from all of the breast cancer patients, it has been provided with the clinical response data, like tumour subclasses and the detection of relapse in receiving surgery. However, GSE1561 did not have clinical response data. The variables of the GSE1456 dataset are Subtype (basal/luminal/ERBB2/normal-like), SURV_RELAPSE (time until relapse/no relapse), and SURV_DEATH (time until death/censoring). Table 3 shows the detection of the multicollinearity diagnostic measures on the GSE1456. The results show that all of the non-significant variables and coefficient(s) may be due to multicollinearity.

### 3.2. Stratified Ten-Fold Cross-Validation within the Datasets

Table 4 compares the AUC values acquired from breast cancer datasets (GSE1561 and GSE1456) using different methods (including eDRW+) in the cross-validation within datasets. The results show that using the logistic regression model and Naïve Bayes in eDRW+ provided the highest AUC values in GSE1456 compared with the other methods. The improved weighting strategy using one-way ANOVA (F-test) can help improve the method’s performance, and is applicable for multiclass classification. For GSE1561, eDRW+ performs better than mean-centering and magnitude-normalization. As a result, the use of pathways in the microarray analysis can serve better than the gene-based classification method. 

Table 5 and Table 6 present the comparative analysis of the different methods for the GSE1561 and GSE1456 datasets in AUC. The tables also state the differences in the AUC values between eDRW+ and the other techniques in terms of percentages. Both tables show the statistically significant differences between eDRW+ and the other methods, as supported by the *p*-values and a 95% confidence interval. The eDRW+ demonstrated consistent and significant improvements over the other methods, with a minimum average difference of 0.65%.

### 3.3. Stratified Ten-Fold Cross-Validation between the Datasets

Table 7 presents the comparison of the AUC values between the methods in a stratified ten-fold cross-validation between the datasets. The table shows the average AUC value of eDRW+ compared with the use of classifiers (e.g., the logistic regression model, SVM, Naïve Bayes). GSE1456 was used as a training set, and GSE1561 was used as a test set. Furthermore, GSE1561 was used as a training set, and GSE1456 was used as a test set. Additional work for stratified ten-fold cross-validation between the datasets using GSE1456 was used as a training set, and GSE19536 was used as a test set. GSE19536 has the same breast cancer subtypes (e.g., luminal, basal, ERBB2, and normal-like) as GSE1456. This dataset has 17,050 genes and 108 samples (luminal: 61; basal: 16; ERBB2: 18; and normal-like: 13) [55,56]. However, the Naïve Bayes used by eDRW+ have better performance than those with the logistic regression model and SVM.

### 3.4. Biological Context Verification and Validation of the Identified Pathways and Genes

Table 8 shows the biological context verification and validation of the pathways and genes identified by eDRW+ using PubMed text data mining and the Functional Annotation Tool from DAVID. The identified pathways and genes were validated based on the literature published in the PubMed and OMIM databases. The OMIM database is an online catalogue of human genes and genetic disorders updated daily. PubMed text data mining can mine the data that automated systematic queries with different keywords. The proposed eDRW+ identified 953 informative genes within 52 informative pathways for the GSE1456 dataset, and 536 within 24 informative pathways for the GSE1561 dataset. The cancer pathway markers and gene markers are referred to as prognostic markers.

Table 9 compares the identified pathways between DRW and eDRW+ for breast cancer. Endocytosis (HSA04144) and the Wnt signalling pathway (HSA04310) have been detected by both DRW and eDRW+ in breast cancer. The Wnt signalling pathway is essential in controlling breast cancer progression [57]. It has been conducted in several clinical trials for breast cancer treatment. The Wnt signalling pathway related to breast cancer involves classification, the immune microenvironment, drug resistance, and molecular agent targeting. The eDRW+ successfully detected the Adherens junction, but not for DRW. It is crucial to regulate tissue integrity and cell dynamics. Therefore, breast cancer development and progression were shown to depend on the malignant destabilization of the adherens junction and the disruption of cell–cell adhesion [58]. 

## 4. Discussion

In previous research, some of the methods for pathway-based microarray analysis were restricted to use in binary class classification, and did not select pathways [11,28,59]. Therefore, eDRW+ has been proposed to identify the prognostic markers in multiclass breast cancer expression data. This method applies one-way ANOVA using an F-test and pathway selection to improve the weight of the genes and increase the classification performance of the method. Besides this, the presence of multicollinearity was a concern in the research. The ANOVA F-test used by the proposed methods can help to reduce the degree of multicollinearity. The ANOVA test can also increase the methods’ sensitivity and AUC, because the AUC is a cut-off point between specificity and sensitivity [60]. The ANOVA test can assist the methods in the identification of differentially expressed genes between multiple classes of samples in order to deal with multiclass problems [19,20,37]. Multiclass classification can identify distinct cancer subtypes in the diagnoses and the decision-making process [27,61]. Some prognoses of patients showing stages of such a disease depend on the thickness of a tumour at the time of surgical treatment [62]. The weight of the genes is essential for the identification of the informative genes in the directed graph, and for use as a measure of the topological importance of genes. Pathway selection can help the methods to select informative pathways and increase their classification performance. Among the use of classifiers, a logistic regression model was used by eDRW+, for a better classification performance. The logistic regression model was a non-parametric and multivariate statistical method for classification [46]. Because there was no assumption for the distribution of the predictor variables, the logistic regression model was relatively robust, and was easily used as a classifier for a meaningful interpretation.

Mean-centering and magnitude-normalization, PCA and AHC were used to analyse the patient’s prognoses and predict their survival accuracy. However, it is insufficient to manipulate gene expression data only to study complex diseases [7,8,59]. Other than gene expression data, pathways also provide a better understanding of molecular mechanisms based on cancer subtypes [27]. NCFS-CORG and NCFS-i use the t-test to classify the cancer datasets into two classes of samples [12,13]. However, the t-test is mainly limited to the classification of two classes of samples [16,17,18,19,20].

A prognostic marker helps to identify a disease outcome, which can be beneficial in cancer treatment and drug discovery [60]. In previous studies, most methods have ignored the analysis of cancer-related markers that can interact in the form of a cancer-related pathway or network [63]. All of the pathways and genes identified by eDRW+ are biologically context-verified and validated using PubMed text data mining and the Functional Annotation Tool. At least seven pathway markers and four gene markers are detected by eDRW+ in breast cancer. The p53 signalling pathway (HSA04115) and mTOR signalling pathway (HSA04150) identified cancer-signalling pathways in this research. Hence, another shift shifted towards the inhibition of the critical cancer-signalling pathways within tumour cells or support cells [64]. These pathways can help increase cancer survival prediction by cancer treatments, including surgery, radiotherapy and chemotherapy. The p53 signalling pathway can provoke apoptosis in response to DNA damage after irradiation in breast cancer [65]. Western blot analysis also showed that the expression level of p53 signalling pathway-related proteins was significantly increased in human breast cancer cell line MCF7. Besides this, the p53 signalling pathway and cell cycle are involved in cell growth and death. The mTOR signalling pathway was significantly involved in the progression of Invasive Lobular Carcinoma (ILC) [66].

Table 10 summarizes the identified genes and pathways annotated by the KEGG and OMIM databases. AKT1 (Gene Entrez ID 207) and TSG101 (Gene Entrez ID 7251) are the breast cancer gene markers that eDRW+ has detected for the GSE1456 and GSE1561 datasets. PIK3CA (5290) and PPM1D (8493) are two breast cancer gene markers in the GSE1561 dataset. The rest of the genes in Table 10 were detected in the GSE1456 dataset. Among the identified genes, CFL1 (Gene Entrez ID 1072) and BRCA2 (Gene Entrez ID 675) were validated as the basal and luminal breast cancer gene markers [67,68]. In the literature, RAD21 (Gene Entrez ID 5885) was validated as a luminal, basal, and ERBB2 breast cancer gene marker [69]. Luminal breast cancer is positive for oestrogen and progesterone receptors (ER and PR), and can express ERBB2 (Gene Entrez ID 2064) [70]. Triple-negative breast cancers (TNBC) lack the expression of ER, PR, and ERBB2 receptors, especially the basal subtype. The Wnt signalling pathway is essential for mammary gland development and breast cancer [71]. The activation of the Wnt signalling pathway is implicated in tumour growth and the poor prognosis of triple-negative breast cancer that lacks the expression of HER2, estrogen, and progesterone receptors. Human amphiphysin 1 is involved in endocytosis, and its expression is increased in breast cancer, but the knockdown of human amphiphysin 1 in breast cancer cells promotes breast cancer progression [72]. With the xenograft mouse model, the silencing of human amphiphysin 1 increased the final breast cancer tumour volume and cell growth.

## 5. Conclusions

The identification of prognostic markers for multiclass cancer expression data has been proposed using eDRW+. eDRW+ has used the ANOVA F-test and pathway selection to improve the weight of the genes, and to identify pathway markers and gene markers. The ANOVA F-test is commonly used to deal with multiclass classification, and pathway selection can select informative pathways. However, the use of the ANOVA F-test influences the calculation of the weight of the genes in the directed graph that is essential to infer a higher reproducibility power of pathway activity. A prognostic marker helps to identify a disease outcome for cancer treatment and drug discovery. Hence, the eDRW+ has successfully identified the cell cycle, p53 signalling pathway, and TP53 gene as cancer markers for breast cancer. Using PubMed text data mining and the Functional Annotation Tool for biological context verification and validation, the TP53 gene was significantly associated with the development and invasion of tumour cells.

The weight of the genes is essential for pathway topology-based microarray analysis that shows the interaction between genes that have connected with each other, especially in the directed graph [4]. Furthermore, the weight strategy used in differential expression analysis assists in the identification of informative genes. The genes with high connectivity and more weight show the better detection of highly connected genes [10]. The Welch test is expected to improve the weight of the genes to solve the multiclass problems instead of using a one-way ANOVA F-test. In previous studies, one-way ANOVA using an F-test represents the general use for multiclass classification. The Welch test can perform much better than the ANOVA F-test [20]. One-way ANOVA assumes that all of the groups share a common variance, and does not consider the difference of the means between the groups. If the groups have unequal variances, the obtained results can be false. Besides this, the suggested test is insensitive to unequal variances and does not assume equal variances. 

## Figures and Tables

**Table 1:** Details of the gene expression data for breast cancer to be studied.

GEO Accession Numbers	Number of Genes (After Pre-Processed)	Number of Samples with Classes	Links
GSE1561 [32]	12,437	Luminal: 27 Basal: 16 Apocrine: 6	www.ncbi.nlm.nih.gov/geo/query/acc.cgi?acc=GSE1561 (accessed on 15 February 2021)
GSE1456 [33]	12,437	Luminal: 62 Basal: 25 ERBB2 ^1^: 15 Normal: 37	www.ncbi.nlm.nih.gov/geo/query/acc.cgi?acc=GSE1456 (accessed on 15 February 2021)

^1^ ERBB2 (Receptor tyrosine–protein kinase erythroblastic oncogene B-2).

**Table 2:** Comparison between DRW and eDRW+.

Comparisons	DRW [4]	eDRW+
Gene expression data	Binary class	Multiclass
Pathways	Metabolic and Non-metabolic Pathways	
Number of genes in the directed graph	4113 genes	
Repeated and missing genes	No	Yes (required for datasets pre-processing)
Normalization based on z-scores	Yes	
Differential expression analysis	*t*-test (equal variances)	One-way ANOVA (F-test)
Restart probability (*r*)	0.7	
Interaction between genes	Interaction between two genes (directed edges)	
Position of genes	Yes	
Weight of the genes	Yes	
Pathway selection	No	Yes

**Table 3:** Detection of the multicollinearity diagnostic measures on the GSE1456.

Main Variable	Other Variables	VIF ^1^	TOL ^2^	Leamer ^3^	Red Indicator ^4^	*R* ^2^ ^5^
Subtype	SURV_RELAPSE	12.68	0.08	0.28	0.63	0.24
	SURV_DEATH	9.16	0.11	0.33		

^1^ VIF (greater than 3, 5 and 10): critical levels of collinearity exist among the regressors. ^2^ TOL and ^3^ Leamer’s method (near to 0): collinearity exists among the regressors. ^4^ Red indicator: absence of redundancy (near to 0) and maximum redundancy (near 1). ^5^ $R^{2}$ (greater than 0.8): a severe problem of multicollinearity.

**Table 4:** Comparison of AUC between the different methods of cross-validation within the datasets.

Method	eDRW+			DRW (Re-Run)	Mean-Centering & Magnitude-Normalization	PCA & AHC	NCFS-CORG		NCFS-i			
Classifier	LR	SVM	NB	LR	CPR	CR	LR	NB	LR	LR (GS)	NB	SVM
GSE1561 [32]	0.95	0.92	0.94	**0.97**	0.80	NA	NA	NA	0.95	NA	0.95	NA
GSE1456 [33]	0.91	0.80	**0.92**	0.91	NA	0.55	0.75	0.83	0.75	0.86	0.84	0.90

Bold values: the highest values. NA: no information and no references cited. LR: Logistic regression model; SVM: Support vector machine; NB: Naïve Bayes; CPR: Cox proportional-hazards regression model; CR: Cox regression model; GS: Genetic search.

**Table 5:** Comparative analysis of the different methods for the GSE1561 dataset in AUC.

eDRW+ (LR) vs. Compared Methods	Mean-Centering & Magnitude-Normalization	NCFS-i
*p*-values	$1.497\times 10^{-10}$	0.0678
95% Confidence Interval	(0.1546, 0.1783)	(−0.0336, 0.0013)
Difference (%)	16.65	0.65

LR: Logistic regression model.

**Table 6:** Comparative analysis of different methods for the GSE1456 dataset in AUC.

eDRW+ (LR) vs Compared Methods	PCA & AHC	NCFS-CORG	NCFS-i (Genetic Search)
*p*-values	$2.200\times 10^{-16}$	$1.169\times 10^{-13}$	$4.896\times 10^{-9}$
95% Confidence Interval	(0.3566, 0.3671)	(0.1586, 0.1691)	(0.0446, 0.0551)
Difference (%)	36.18	16.38	4.98

LR: Logistic regression model.

**Table 7:** Comparison of the AUC between the different methods in the cross-validation between the datasets.

Training Set	GSE1456	GSE1456	GSE1561
Test Set	GSE1561	GSE19536	GSE1456
eDRW+ (Logistic regression model)	**0.82**	0.80	0.68
eDRW+ (Support vector machine)	0.54	0.53	0.49
eDRW+ (Naïve Bayes)	0.76	**0.81**	**0.69**

Bold values: the highest values.

**Table 8:** Biological context verification and validation on the identified pathways and genes.

Datasets	Number of Identified Pathways	Number of Cancer Pathway Markers		Number of Identified Genes	Number of Cancer Gene Markers	
		PubMed	DAVID		PubMed	DAVID
GSE1561	24	18	7	536	144	4
GSE1456	52	39	13	953	315	9

PubMed: PubMed text data mining (refers to the PubMed database). DAVID: Functional Annotation Tool from DAVID (refers to the OMIM database). Refer to Supplementary Table S1 for more details.

**Table 9:** Comparison of the cancer pathway markers between DRW and eDRW+.

KEGG Pathway IDs	Pathway Names	DRW	eDRW+
HSA04010	MAPK signaling pathway	Yes	No
HSA04020	Calcium signaling pathway	Yes	No
HSA04144	Endocytosis	Yes	Yes
HSA04310	Wnt signaling pathway	Yes	Yes
HSA04520	Adherens junction	No	Yes
HSA04810	Regulation of actin cytoskeleton	Yes	No

Yes: the pathway has been detected. No: the pathway has not been detected.

**Table 10:** Identification of the genes and pathways annotated by the KEGG and OMIM databases.

Gene Entrez ID	KEGG Pathway IDs	OMIM_DISEASE
207 (AKT1)	HSA04150: mTOR signalling pathway, HSA04620: Toll-like receptor signalling pathway, HSA04630: Jak-STAT signalling pathway, HSA04914: Progesterone-mediated oocyte maturation, HSA04920: Adipocytokine signalling pathway	Breast cancer, Colorectal cancer, Ovarian cancer
7251 (TSG101)	HSA04144: Endocytosis	Breast cancer
367 (AR)	HSA04114: Oocyte meiosis	Prostate cancer, Breast cancer
675 (BRCA2)	HSA03440: Homologous recombination	Breast cancer, Prostate cancer, Pancreatic cancer
841 (CASP8)	HSA04115: p53 signalling pathway, HSA04620: Toll-like receptor signalling pathway, HSA04622: RIG-I-like receptor signalling pathway	Breast cancer, Hepatocellular carcinoma, Lung cancer
999 (CDH1)	HSA04520: Adherens junction	Breast cancer, Gastric cancer, Ovarian cancer, Prostate cancer
5888 (RAD51)	HSA03440: Homologous recombination	Breast cancer
7157 (TP53)	HSA04110: Cell cycle, HSA04115: p53 signalling pathway, HSA04310: Wnt signalling pathway	Breast cancer, Colorectal cancer, Hepatocellular carcinoma, Pancreatic cancer, Nasopharyngeal carcinoma, Basal cell carcinoma
11,200 (CHEK2)	HSA04110: Cell cycle, HSA04115: p53 signalling pathway	Breast cancer, Prostate cancer, Colorectal cancer
5290 (PIK3CA)	HSA04070: Phosphatidylinositol signalling system, HSA04150: mTOR signalling pathway, HSA04660: T cell receptor signalling pathway, HSA04910: Insulin signalling pathway	Breast cancer, Colorectal cancer, Ovarian cancer, Non-small cell lung cancer, Hepatocellular cancer, Gastric cancer
8493 (PPM1D)	HSA04115: p53 signalling pathway	Breast cancer

## Data Availability

The data analyzed in this paper are available in the Gene Expression Omnibus (GEO) repository at NCBI (www.ncbi.nlm.nih.gov/geo/query/acc.cgi?acc=GSE1561; www.ncbi.nlm.nih.gov/geo/query/acc.cgi?acc=GSE1456) (accessed on 15 February 2021).

## Supplementary Materials

The following are available online at https://www.mdpi.com/article/10.3390/e23091232/s1, Table S1: Lists of identified genes and pathways using eDRW+. Table S2: The identified genes and pathways annotated by the KEGG and OMIM databases, based on the GSE1456 dataset. Table S3: The identified genes and pathways annotated by the KEGG and OMIM databases, based on the GSE1561 dataset. Table S4: List of the identified pathways and genes from the GSE1456 dataset, with its *p*-values. Table S5: List of the identified pathways and genes from the GSE1561 dataset, with its *p*-values.
